# Efficacy of pine leaves as an alternative bedding material for broiler chicks during summer season

**DOI:** 10.14202/vetworld.2015.1219-1224

**Published:** 2015-10-22

**Authors:** Gourav Sharma, Asma Khan, Surender Singh, Ashok Kumar Anand

**Affiliations:** Division of Livestock Production and Management, Faculty of Veterinary Sciences and Animal Husbandry, Sher-e-Kashmir University of Agricultural Sciences & Technology of Jammu, R.S.Pura, Jammu - 181 102, Jammu and Kashmir, India

**Keywords:** litter material, paddy husk, paddy straw, pine leaves

## Abstract

**Aim:**

The aim was to assess the efficacy of pine leaves as an alternative bedding material on the performance of broiler chicks.

**Materials and Methods:**

The present study was conducted in summer. Total 120, day old Vencobb straight run chicks were procured, and after 5 days of brooding, chicks were randomly distributed into four treatment groups viz. paddy husk (Group I), paddy straw (Group II), pine leaves (Group III), and combination of paddy straw and pine leaves (Group IV), each having 30 chicks with 3 replicates of 10 chicks each. Chicks were reared under intensive conditions in houses that have a semi-controlled environment, with optimum temperature and adequate ventilation. Food and water were provided as per NRC (1994) requirement.

**Results:**

The average body weight after 6 weeks of the experiment was 2018.83±31.11, 1983.80±33.27, 2007.36±35.73, and 1938.43±36.35 g. The bedding type had no significant effect on the carcass characteristics viz. evisceration rate and proportion of cut-up parts of the carcass except giblet yield. The experiment suggested that performance of broiler chicks reared on paddy straw and pine leaves as litter material, had improved body weight and feed conversion ratio as compared to rearing on paddy husk as bedding material. Bacterial count, parasitic load and the N, P, K value of manure of different bedding material shows no significant difference.

**Conclusion:**

Pine leaves have a potential to be used as an alternative source of litter material to economize poultry production in a sustainable way, so as to make poultry farming as a profitable entrepreneur.

## Introduction

The poultry industry is one of the largest and fastest growing sectors of livestock production in the world with a 35% increase in meat and egg production in the period from 2000 to 2008 [1]. The 2010 world annual census data estimated the world flock to be over 18 billion birds with an estimated yearly output of 22 million tons of manure [1]. The deep litter system is the most popular system of housing in broiler production throughout the world. A variety of paper products [2], gypsum [3], hardwood bark [4], kenaf [5], peanut hulls [6], sand [7,8], rice hulls [9], rice hull ash [10], rice and wheat straw [11], and soft wood chipping fines [12] have occasionally been used as substitute bedding materials with various degree of success. Total replacement of the litter after every flock results in considerable environmental impact due to the high amounts of substrate required (e.g., wood shavings, straw, or sawdust), and the destination of this residue in the environment [13-15]. Therefore, the selection of litter material type, quantity and quality and availability of that have an important role in controlling environment within poultry house and bird performance [16]. Chicken litter is a mixture of feces, wasted feeds, bedding materials, and feathers [17,18]. Litter type affects litter consumption and litter bacteria [2], thus may affect body weight and carcass characteristics of broiler chicks. The three wastes of primary concern in poultry production are the bedding materials used for poultry housing, the manure resulting from poultry production and dead birds, common to all operations [19].

Litter material play a crucial role in the rearing of poultry. Common litter material used in the poultry industry includes wood shavings, sawdust, sand, pine shaving, shredded papers or paper chips, dry straw, rice hulls, maize cobs, corn silage; peat has alternative litter materials [20,21]. Pine shavings have been the preferred material for broiler production in the southeastern US for many years [22]. About 60% of farmers use saw dust as the bedding material, 16.7% use wood chippings, 20% use both wood chipping and saw dust while 3.3% of farmers do not use bedding materials. The type of bedding material used by farmers depends on locality and availability [23]. Paddy husk is most preferred and commonly used bedding source for poultry in northern India. In recent year, paddy husk has extensively used material by other industries as raw material for biofuel. However, due to diversified use of these materials, their availability is decreasing, and cost is increasing day by day. With the banning of cage system by EU and launching of National Program for Organic Production [24] in India, requirement of bedding material is expected to increase many folds in near future and make the poultry industry less profitable entrepreneur. Therefore, it is necessary to seek alternative sources of bedding material to make the poultry industry as a sustainable entrepreneur. State like J & K is blessed with pine forests (1825 km^2^) as per Directorate of Economics and Statistics [25] which could be exploit in poultry production by utilizing its wood shaving [26] or pine leaves as litter material in a sustainable way.

The pine leaves are utilizing as a bedding material in pet animals in many countries because of its anti-microbial property and could be a good litter material for raising of poultry. Keeping this in view an experiment was conducted to compare the efficacy of paddy husk, paddy straw, and pine leaves as a litter material for raising of broilers.

## Materials and Methods

### Ethical approval

Permission of the Institutional Animal Ethics Committee was taken prior to the start of the experimental study.

### Location and preparation of poultry shed

The experiment was carried out at the Poultry shed having semi-controlled environment, with optimum temperature and adequate ventilation, SKUAST-J, R. S. Pura, Jammu is located at 32°38′N 74°44′ E/32.63°N 74.73°E at an average elevation of 270 m (886 ft) during the summer season (10^th^ May-21^st^ June, 2013). The pens were cleaned and disinfected with phenyl solution before spreading litter and placing birds at random.

### Collection and processing of bedding material

The bedding materials used were collected from a local area of Jammu region in the month of December-March 2013. After proper drying under the sun, they were processed for the preparation of bedding material. The bedding materials were provided with a depth of 5 cm during the rearing period.

### Experimental design

The experiment was conducted in completely randomized design, a total of 120, day-old commercial (Vencobb) broiler chicks were procured and after 5 days of brooding were randomly divided into four groups having total 30 chicks in each group with their 3 replicates, each having 10 chicks and kept in a deep litter system. The litter material used was paddy husk (Group I), paddy straw (Group II), pine leaves (Group III), and (Group IV) with combination of paddy straw and pine leaves (50:50). The ration fed to the broiler birds were procured from commercial out let having nutritive value mentioned in Table-1 and other managerial conditions were similar throughout the study period.

**Table-1:** Nutritive composition of broiler feed (percentage on dry matter basis).

Proximate principles	Broiler starter	Broiler grower	Broiler finisher
CP (%)	22.03	20.24	18.06
ME (kcal/kg feed)	2858	2895	2960
C:P Ratio	129.73	143.03	163.89

CP=Crude protein, ME=Metabolizable energy, C:P=Calorie:Protein

### Meteorological parameters

The overall mean maximum temperature recorded inside the poultry shed ranged from 33.75°C to 43.42°C (overall mean - 38.39°C) and outside the poultry shed ranged from 34.18°C to 43.05°C (overall mean - 38.40°C), and the overall mean minimum temperature recorded in the poultry shed ranged from 22.81°C to 29.86°C (overall mean - 26.13°C) and outside the poultry shed ranged from 22.57°C to 29.48°C (overall mean - 25.50°C). The percent relative humidity ranged from 28.61% to 68.42% in the morning (overall mean - 46.58%), 47.15-52.21% in the afternoon (overall mean - 49.47%) and 52.24-67.14% in the evening (overall mean - 60.52%). The high and low percent relative humidity outside the shed was ranged from 27.57% to 63.71% (overall mean - 44.26%) and 12.14-50.14% (overall mean - 31.71%), respectively. The mean temperature humidity index (THI) varied from 71.10 to 76.21 (overall mean - 74.02) during morning (0900 h), 87.44-88.87 (overall mean - 88.01) during afternoon (1300 h), and 75.34-81.20 (overall mean - 77.99) during evening (1800 h).

### Data recording

The data pertaining to body weights, feed consumption, carcass characteristics, bacterial and parasitic load, moisture content and temperature, relative humidity, and THI were collected up to 6 weeks of age. At the end of the experiment, litter samples were collected and examined for manorial value by estimating the nitrogen (N), phosphorus (P) content [27] and potassium (K) content by flame photometer. Ammonia estimation was also conducted at end of the experiment [28].

### Statistical analysis

The results obtained were subjected to analysis of variance and treatment means were ranked using Duncan’s multiple range test. Significance was declared at p<0.05 unless otherwise stated.

## Results

The performance of broilers on different bedding types are summarized in Table-2. At the age of 42 days, body weight of broilers was not significantly (p<0.05) affected by the litter type. Broilers grown in Group I had the highest body weight and Group IV had the lowest body weight (Table-3). Accordingly, body weight gain per day was highest in Group I and lowest in Group IV. Total feed intake in different groups of broilers did not differ significantly. Broilers reared on Group I have highest dry matter feed intake whereas those reared on Group IV have lowest dry matter feed intake (Table-4). Feed conversion ratio (FCR) of Group I and Group III did not differ significantly and were lower when compared to other two groups. Percentage of eviscerated weight, breast, and thigh were not affected by litter type (p<0.05), but significant differences were observed for giblet yield (Table-5). Percentage of eviscerated weight and breast were highest in Group I and lowest in Group IV and percentage of the thigh was highest in Group II and lowest in Group III. Percentage of giblet yield was significantly different and tended to be highest in Group I and lowest in Group II.

**Table-2:** Overall mean±SE of growth performance of chicks.

Parameters	Treatment			

	Group I	Group II	Group III	Group IV
Average body weight (g)	2018.83±31.11	1983.80±33.27	2007.36±35.73	1938.43±36.35
Body weight gain (g)	1930.47±7.83	1895.33±5.34	1917.43±9.43	1848.93±4.38
Feed intake (g)	3623.47±28.15	3604.33±32.66	3607.65±33.48	3588.63±26.86
FCR	1.88±0.02	1.90±0.04	1.88±0.02	1.94±0.03

Group I=Chicks reared on paddy husk, Group II=Chicks reared on paddy straw, Group III=Chicks reared on pine leaves, Group IV=Chicks reared on combination of paddy straw and pine leaves, FCR: Feed conversion ratio, SE=Standard error

**Table-3:** Mean±SE of weekly body weight of chicks.

Age (weeks)	Body weight (g/bird)			

	Group I	Group II	Group III	Group IV
1	258.37±4.02	250.50±3.98	257.33±4.37	260.60±3.75
2	478.80±6.32	474.47±8.11	496.07±7.61	476.60±7.32
3	735.13±11.44	711.03±13.33	731.20±12.68	720.83±13.63
4	1036.00±20.15	1053.93±21.84	1073.86±19.85	1064.50±18.79
5	1516.90±27.71	1441.20±25.87	1500.10±24.21	1428.40±21.68
6	2018.83±31.11	1983.80±33.27	2007.36±35.73	1938.43±36.35

Group I=Chicks reared on paddy husk, Group II=Chicks reared on paddy straw, Group III=Chicks reared on pine leaves, Group IV=Chicks reared on combination of paddy straw and pine leaves, SE=Standard error

**Table-4:** Mean±SE of weekly feed intake of chicks.

Age (weeks)	Weekly feed intake (g/bird)			

	Group I	Group II	Group III	Group IV
1	179.13±1.16	177.76±0.81	180.13±1.09	180.17±0.27
2	359.27±12.14	358.73±14.97	358.50±10.56	347.70±12.71
3	449.4±10.85	453.47±6.95	455.70±6.96	447.50±4.90
4	653.67±13.56	668.50±7.85	653.13±2.69	670.47±6.46
5	931.50±7.39	892.37±36.48	910.33±14.77	890.63±12.26
6	1050.50±5.79	1053.50±3.27	1049.86±7.76	1052.16±3.91

Group I=Chicks reared on paddy husk, Group II=Chicks reared on paddy straw, Group III=Chicks reared on pine leaves, Group IV=Chicks reared on combination of paddy straw and pine leaves, SE=Standard error

**Table-5:** Effect of litter type on evisceration rate and proportion of premium parts of the carcass.

Parameters (%)	Carcass characteristics			

	Group I	Group II	Group III	Group IV
Eviscerated WT^1^	65.16±1.04	63.98±0.80	64.38±0.90	62.70±0.44
Breast^2^	32.72±1.57	29.92±0.24	31.67±0.51	28.47±1.82
Thigh^2^	16.87±0.28	17.74±0.29	16.18±0.92	17.62±0.43
Giblet	7.21±0.04^a^	4.37±0.08^a,b^	7.18±0.03^a^	5.78±0.66^b^

a,b Mean values bearing different superscripts in a row differ significantly (p<0.05). Group I=Chicks reared on paddy husk, Group II=Chicks reared on paddy straw, Group III=Chicks reared on pine leaves, Group IV=Chicks reared on combination of paddy straw and pine leaves. Note: Expressed as

(^1^) per cent of body weight,

(^2^) per cent of eviscerated weight

The bacterial load (*Staphylococcus* and *Escherichia coli* counts) and parasitic load were slightly higher in Group IV and lower in Group III showing a non-significant difference (Table-6). The data recorded for N, P, and K content of the manure in the experiment (Table-7) indicated that there was no significant difference in N, P, and K content of the litter among various groups. The nitrogen content was higher in Group III and lowest in Group I, the phosphorus content was higher in Group III and lowest in Group II and the potassium content was higher in Group III and lowest in Group II. Ammonia concentration was calculated by following the procedure as employed by Moum *et al*.[28]. For the purpose of ammonia estimation, each pen was covered with a transparent plastic sheet. A pH paper strip having a range of 6-7.7 and 9.5, moistened with sterile neutral water was held for 15 s at the level of birds in each pen. It was found that the ammonia production was similar in all the treatment groups and all the treatment groups show a change in orange color of the pH paper strip which indicates that the ammonia concentration in the different treatment groups was between 25 and 50 ppm (parts per million).

**Table-6:** Overall mean±SE of the bacterial and parasitic load.

Parameters	Group I	Group II	Group III	Group IV
*Staphylococcus*×10^9^/ml	1.25±0.05	1.27±0.06	1.23±0.05	1.31±0.05
*E. coli*×10^10^/ml	2.50±0.24	2.98±0.19	2.33±0.14	3.45±0.10
Parasitic count (oocysts/field)	19.21±3.16	20.39±3.82	17.01±2.18	21.06±2.06

Group I=Chicks reared on paddy husk, Group II=Chicks reared on paddy straw, Group III=Chicks reared on pine leaves, Group IV=Chicks reared on combination of paddy straw and pine leaves, *E. coli=Escherichia coli*, SE=Standard error

**Table-7:** Overall mean±SE of nitrogen, phosphorus and potassium contents of litter.

Manurial values	Treatment			

	Group I	Group II	Group III	Group IV
Nitrogen (N_2_), %	2.03±0.37	2.11±0.23	2.86±0.31	2.29±0.27
Phosphorus (P_2_O_5_), %	1.83±0.09	1.81±0.07	1.92±0.06	1.84±0.04
Potassium (K_2_O), %	1.43±0.06	1.39±0.05	1.47±0.05	1.42±0.03

Group I=Chicks reared on paddy husk, Group II=Chicks reared on paddy straw, Group III=Chicks reared on pine leaves, Group IV=Chicks reared on combination of paddy straw and pine leaves, SE=Standard error

## Discussion

In the present study, broilers reared in Group IV had the lowest body weight in comparison to Group I, II, and III (Table-2). The observed differences in body weight may be attributed to depression of feed intake in birds reared in Group IV and increasing feed intake on others litter especially on paddy husk (Table-3 & 4). Our result were in agreement with the finding of Davis *et al*. [21] that the type of litter material had no significant effect on body weight of broiler and Navneet *et al*. [29] observed that paddy straw can also be used as an alternate bedding material for broilers. The findings of Mahmoud *et al*. [30], and Farhadi, [31] that litter type have significant effect on body weight at 4, 5, and 6 weeks of age and rice hulls due to its favorable properties could be successfully used as alternative poultry litter material were contradictory with the result. Many studies, in which alternative materials were tested, have reported that the type of litter material used does not affect body weight [31]. FCR of broilers was not affected by litter type (Table-2). Other researchers have reported similar findings regarding the influence of various litter material on FCR [3,32-34]. The bedding type had no influence on the carcass characteristics viz. evisceration rate and proportion of cut-up parts of the carcass except the giblet yield. Our results were in accordance with Atapattu and Wickramasinghe [34], and Grimes [35] who also did not find any significant effect of the type of litter on the carcass characteristics.

The bacterial load were slightly higher in Group IV and lower in Group III showing a non-significant difference (Table-6) which is similar to Estevez [36] who also observed higher incidence of *E. coli* infection in a combination of ammonia and wet litter. The coccidial load in Group IV was higher and lower in Group III. Coccidial infection was mainly influenced by the moisture content of the litter. Moisture content was highest in Group IV (25.69±1.79) and lowest in Group III (22.53±1.40). Waldenstedt *et al.*, [37] also reported that the frequency of outbreaks of coccidiosis in chicks reared under damp and moist conditions was higher.

The nitrogen content reported in our finding has no significant difference among various litter material which was consistent with the Biswas *et al.*, [38] who also reported no significant effect of bedding material viz. sugarcane bagase, sawdust and wheat straw on the N content of the litter. Whereas K and P contents in wheat straw were reported to be significantly higher as compared with other litter materials which may be due to the difference in the initial content of these nutrients in the type of bedding materials used by these workers. However, our result was contradicted in relation to P and K content in litter material.

## Conclusion

In conclusion, it may be stated that paddy straw and pine leaves could be used as an alternate litter to commonly used paddy husk to promote the economical intensive poultry production system.

## Authors’ Contributions

GS was the MVSC scholar of the division who carried out experimental research work and laboratory analysis of data. AK was guide of GS, under whose supervision, the thesis was submitted. AK planned and designed the experiment; provide needful guidance to execute the proposed experimental design, executed statistical analysis of data, drafted and revised the manuscript as per journal format. AK was head of the division. SS and AKA help in evaluating the proximate principle and manorial value of various litter samples. All authors read and approved the final manuscript.

## References

[1] FAO (2010). Agricultural Handbook: Poultry Meat and Eggs.

[2] Lien R.J., Conner D.E., Bilgili S.F. (1992). The use of recycled paper chips as litter material for rearing broiler chickens. Poult. Sci.

[3] Grimes J.L., Carter T.A., Godwin J.L. (2006). Use of a litter material made from cotton waste, gypsum, and old newsprint for rearing broiler chickens. Poult. Sci.

[4] Brake J.D., Boyle C.R., Chamblee T.N., Schultz C.D., Peebles E.D. (1992). Evaluation of the chemical and physical properties of hardwood bark used as a broiler litter material. Poult. Sci.

[5] Malone G.W., Tilmon E.D., Taylor R.W. (1990). Evaluation of kenaf core for broiler litter. Poult. Sci.

[6] Lien R.J., Hess J.B., Conner D.E., Wood C.W., Shelby R.A. (1998). Peanut hulls and a litter source for broiler breeder replacement pullets. Poult. Sci.

[7] Billgilli S.F., Montenegro G.I., Hess J.B., Eckman M.K. (1999a). Sand as litter for rearing broiler chickens. J. Appl. Poult. Res.

[8] Shields S.J., Garner J.P., Mench J.A. (2005). Effect of sand and wood shavings bedding on the behavior of broiler chickens. Poult Sci.

[9] Veltmann L.R., Cardoer F.A., Unlon S.S. (1984). Comparison of rice hull products as litter material and dietary fat levels on turkey poult performance. Poult. Sci.

[10] Chamblee T.N., Yeatman J.B. (2003). Evaluation of rice hull ash as broiler litter. J. Appl. Poult. Res.

[11] Benabdeljelil K., Ayachi A. (1996). Evaluation of alternative litter materials for poultry. J. Appl. Poult. Res.

[12] Parsons A.H., Baker S.L. (1985). Softwood chipping fines efficacy as poultry litter. Poult. Sci.

[13] Pandey P.K., Soupir M.L. (2011). *Escherichia coli* inactivation kinetics in anaerobic digestion of dairy manure under moderate, mesophilic and thermophilic temperatures. AMB Express.

[14] Pote D.H., Way T.R., Kleinman P.J., Moore P.A., Meisinger J.J., Sistani K.R., Saporito L.S., Allen A.L., Feyereisen G.W. (2011). Subsurface application of poultry litter in pasture and no-till soils. J. Environ. Qual.

[15] Watts D.B., Way T.R., Torbert H.A. (2011). Subsurface application of poultry litter and its influence on nutrient losses in runoff water from permanent pastures. J. Environ. Qual.

[16] Garces A., Afonso S.M.S., Chilundo A., Jairoce C.T.S. (2013). Evaluation of different litter materials for broiler production in a hot and humid environment: 1. Litter characteristics and quality. J. Appl. Pout. Res.

[17] Wilkinson K.G., Tee E., Tomkins R.B., Hepworth G., Premier R. (2011). Effect of heating and aging of poultry litter on the persistence of enteric bacteria. Poult. Sci.

[18] Kim J., Diao J., Shepherd M.W., Singh R., Heringa S.D., Gong C., Jiang X. (2012). Validating thermal inactivation of *Salmonella* spp. in fresh and aged chicken litter. Appl. Environ. Microbiol.

[19] Thyagarajan D., Barathi M., Sakthivadivu R. (2013). Scope of poultry waste utilization. IOSR J. Agric. Vet. Sci.

[20] Swain B.K., Sundaram R.N.S. (2000). Effect of different types of litter material for rearing broilers. Br. Poult. Sci.

[21] Davis J.D., Purswell J.L., Columbus E.P., Kiess A.S. (2010). Evaluation of chopped switchgrass as a litter material. Int. J. Poult. Sci.

[22] Beri M.T. (2011). Choice of litter material can decrease salmonella in poultry flocks. World Poult. J.

[23] Kalu E. (2015). Poultry litter/manure management practices in intensively managed poultry farms in portharcourt. IOSR J. Agric. Vet. Sci.

[24] National Programme for Organic Production, (NPOP) (2005). Ministry of Commerce.

[25] (2008). Government of J&K Directorate of Economics and Statistics. DFO Forest Statistics.

[26] Asaniyan E.K., Agbede J.O., Laseinde E.A.O. (2007). Impact assessment of different litter depths on the performance of broiler chickens raised on sand and wood shaving litters. World J. Zool.

[27] AOAC (2000). Offical Methods of Analysis. Association of Official Analytical Chemists.

[28] Moum S.G., Seltzer W., Goldhaft T.M. (1969). Sampling and measurement of ammonia concentration at animal facility. Poult. Sci.

[29] Navneet K., Nagra S.S., Daljeet K., Hanah S.S. (2011). Paddy straw as an alternate bedding material for broiler chicks. J. World's Poult. Res.

[30] Mahmoud M.S.H., Soliman F.N.K., Bahie-EL-Deen M., EL Sebai A.A. (2014). Effect of different types of litter on broiler performance. Res. J. Poult. Sci.

[31] Farhadi D. (2014). Evaluation of the physical and chemical properties of some agricultural wastes as poultry litter material. Glob. J. Anim. Sci. Res.

[32] Burke G.B., Pescatore A.J., Cantor A.H., Straw M.L., Johnson T.H. (1993). Newspaper as litter material and its effect on the performance of broilers. J. Appl. Poult. Res.

[33] Willis W.L., Murray C., Talbot C. (1997). Evaluation of leaves as a litter material. Poult. Sci.

[34] Atapattu N.S.B., Wickramasinghe K.P. (2007). The use of refused tea as liter material for broiler chicken. Poult. Sci.

[35] Grimes J. (2004). Alternative litter material for growing poultry. North Carolina Poultry Industry Newsletter.

[36] Estevez I. (2002). Ammonia and poultry welfare. Poult. Perspect.

[37] Waldenstedt L., Elwinger K., Lunden A., Thebo P., Uggla A. (2001). Sporulation of *Eimeria maxima* oocysts in litter with different moisture contents. Poult. Sci.

[38] Biswas S.K., Wahid M.A., Karim M.J., Parmanik M.A.H., Rokonuzzaman M. (2001). Evaluation of different materials for broiler performance, coccidial oocyst population and level of N, P and K during winter. Pak. J. Biol. Sci.

